# Coronary microcirculatory dysfunction in hypercholesterolemic patients with COVID-19: potential benefit from cholesterol-lowering treatment

**DOI:** 10.1080/07853890.2023.2199218

**Published:** 2023-04-17

**Authors:** Alpo Vuorio, Petri T. Kovanen, Frederick J. Raal

**Affiliations:** a Forensic Medicine, Mehiläinen Airport Health Centre, Vantaa, Finland; b Department of Forensic Medicine, University of Helsinki, Helsinki, Finland; c Atherosclerosis Research Laboratory, Wihuri Research Institute, Helsinki, Finland; d Faculty of Health Sciences, University of Witwatersrand, Johannesburg, South Africa

**Keywords:** Coronary microcirculatory dysfunction, lipid-lowering drugs, statins, PCSK9 inhibitors, COVID-19, endothelial dysfunction

## Abstract

Patients with hypercholesterolemia often have coronary microvascular dysfunction (CMD). Viral infections, such as the SARS-CoV-2 infection, may also result in CMD. Three non-randomized studies have shown significant beneficial effects of statins on CMD in non-infected patients. Similarly, in SARS-CoV-2 - infected patients one beneficial mechanism of action of statins may be the amelioration of endothelial dysfunction, which is a major driver of CMD. Apart from statins, lipoprotein apheresis and PCSK9 inhibitors can also improve or even reverse CMD. The potential reversal of CMD by using effective cholesterol-lowering medications during and after COVID-19 infection, especially in hypercholesterolemic COVID-19 patients, is important.

**KEY MESSAGES**
- Coronary microvascular dysfunction (CMD) is common in patients hospitalized with SARS-CoV-2 infection
- Three nonrandomized studies in non-infected patients are showing the beneficial effects of statin treatment on CMD
- Effective cholesterol-lowering medication during and after SARS-CoV-2 infection, especially in hypercholesterolemic COVID-19 patients, is of great significance

## Introduction

Coronary microcirculation refers to the coronary vessels with a diameter of less than 500 µm which, in individuals without epicardial atherosclerosis, vasodilate during exercise and thereby augment myocardial perfusion [1]. Coronary microvascular dysfunction (CMD) may lead to impaired coronary flow reserve and a high index of coronary microcirculatory resistance [2–4]. Typical diseases that lead to myocardial ischemia without epicardial coronary atherosclerosis are hypertrophic cardiomyopathy, aortic stenosis, and myocarditis [5–7]. It has been shown that endothelial dysfunction is a key component of CMD pathogenesis [8–10]. By causing endothelial dysfunction, severe hypercholesterolemia can contribute to CMD, and, in a swine model of familial hypercholesterolemia, it was demonstrated that CMD is a contributor to cardiac ischemia even before the development of critical coronary atherosclerotic cardiovascular disease (ASCVD) [9]. Importantly, several human studies have shown that severe hypercholesterolemia, such as in subjects with familial hypercholesterolemia, induces systemic endothelial dysfunction, which often includes CMD [11–14].

## Discussion

In addition to hypercholesterolemia, viral infections may also result in CMD. More than a decade ago CMD was reported in patients with acute respiratory distress syndrome and influenza A (H1N1) [15]. This year an echocardiographic coronary flow velocity reserve analysis revealed that CMD is common in patients hospitalized with SARS-CoV-2 infection [16]. In a very comprehensive study of patients who had recently recovered from COVID-19, patients with classic hypertrophic cardiomyopathy and healthy controls were compared using vasodilator stress cardiovascular magnetic resonance examination for the evaluation of myocardial perfusion reserve (MPR) [17]. In this study significantly reduced MPR was found in the COVID-19 patients compared to healthy controls, and the reduced MPR observed was as severe as that found in the patients with hypertrophic cardiomyopathy. Drakos et al. [17] postulated that the reduced MPR was caused by both direct and triggered mechanisms caused by the SARS-CoV-2 virus infection, which thereby resulted in CMD. This conclusion is supported by a recent review suggesting a variety of injury mechanisms causing endothelial dysfunction in the microcirculatory environments in COVID-19 patients [18].

As statins improve endothelial dysfunction and can reduce vascular inflammation, they were included in the list of secondary prevention strategies depicted in the recent European Society of Cardiology Working Group on Coronary Pathophysiology and Microcirculation position paper on "coronary microvascular dysfunction in cardiovascular disease" [1]. It is noteworthy that, in observational studies statins have been shown to reduce both the morbidity and mortality of patients with SARS-CoV-2 infection and that several similar randomized studies are currently underway [19–21]. One beneficial mechanism of the action of statins may be the reduction of CMD exacerbation caused by the SARS-CoV-2 infection. This opinion is supported by the findings obtained in several nonrandomized studies performed on non-infected patients and which are showing significant beneficial effects of treatment with statins on CMD [22]. The benefit of rosuvastatin was shown in patients with hypertension [23,24], the benefit of atorvastatin in patients with angina pectoris [25], and the benefit of simvastatin in patients with documented ASCVD [26].

Apart from statins, lipoprotein apheresis and PCSK9 inhibitors as cholesterol-lowering strategies can also reverse CMD [27,28]. The benefit of the PCSK9 inhibitor evolocumab was shown in a recent randomized study of 60 hospitalized patients with severe SARS-CoV-2 infection [29]. Within 30 days of observation, the patients treated with a 140 mg subcutaneous injection of evolocumab had lower mortality and less need for intubation compared to the placebo group (23.3 vs. 53.3%; 95% CI −53.4 to − 6.59%). It has been shown earlier that in Dengue virus infection, a PCSK9 inhibitor could interfere with the production of IL-6, thereby reducing the inflammatory response [30]. In addition, the favorable mechanisms of PCSK9 inhibitors, especially in HeFH, could be argued to also result from an effective reduction in the levels of LDL-C and to a lesser extent in the reduction of Lp(a) levels, with a resultant improvement in endothelial function [31,32].

Regarding SARS-CoV-2–infected patients, the microvascular angiopathic consequences on the myocardium may lead to myocardial ischemia and dysfunction with grave consequences [33].

Therefore, it is of paramount importance to aim at preventing the development of CMD in COVID-19 patients.

## Conclusion

Both severe hypercholesterolemia and SARS-CoV-2 infection cause endothelial dysfunction and the associated CMD. Therefore, it is appropriate to attempt to mitigate or reverse CMD in hypercholesterolemic COVID-19 patients by using effective cholesterol-lowering medications, particularly statins. This reminder is particularly important since many hypercholesterolemic patients, including those with familial hypercholesterolaemia in whom the hypercholesterolemia can be severe, remain undertreated or even untreated before, during, and after the infection.

## Data Availability

All data relevant to the study are included in the article.
